# Statistical analysis of the impact of *F*_*e*_*O*_*3*_ and *Z*_*n*_*O* nanoparticles on the physicochemical and dielectric performance of monoester-based nanofluids

**DOI:** 10.1038/s41598-023-39512-9

**Published:** 2023-07-29

**Authors:** Jean Lambert Jiosseu, Asse Jean-Bernard, Ghislain Mengata Mengounou, Emeric Tchamdjio Nkouetcha, Adolphe Moukengue Imano

**Affiliations:** 1 Pure Physique Laboratory UFD MIP, University of Douala, Douala, Cameroon; 2 Laboratory of Technology and Applied Sciences, University of Douala, Douala, Cameroon

**Keywords:** Energy science and technology, Engineering, Nanoscience and technology, Physics

## Abstract

This article deals with a comparative study of the physicochemical and electrical properties of monoesters of castor oil compared with their counterparts based on *FeO*_*3*_ and *Z*_*n*_*O* nanoparticles. The results are also compared with those in the literature on triesters, and also with the recommendations of the IEEE C 57.14 standard. The data is analysed statistically using a goodness-of-fit test. The analysis of the viscosity data at 40 °C shows an increase in viscosity. For concentrations of 0.10 wt%, 0.15 wt% and 0.20 wt% these are respectively 5.4%, 9.69%, 12.9% for *F*_*e*_*O*_*3*_ NFs and 7.6%, 9.91% and 12.7% for *Z*_*n*_*O* NFs. For the same concentrations, the increase in acid number is respectively 3.2%, 2.9%, 2.5% for FeO3 samples and 3.18%, 2.0%, 1.2% for *Z*_*n*_*O* samples. For the same concentrations, the fire point shows an increment of 4%, 3% and 2% for *F*_*e*_*O*_*3*_ samples and a regression of 8.75%, 6.88% and 5.63% for *Z*_*n*_*O* samples. As for the breakdown voltage, for the same concentrations we observe respectively an increment of 43%, 27%, 34% for the *F*_*e*_*O*_*3*_. The results show an improvement on partial discharge inception voltage with FeO3 of 24%, 8.13% and 15.21% respectively for the concentrations 0.10 wt%, 0.15 wt% and 0.20 wt%.

## Introduction

A large majority of power transformers have cellulose (paper/compressed cardboard) and oil insulation. The cellulose part of this insulation has three main functions. Firstly, it insulates the main components of the transformer from the various voltages, storing the electrical charge when the transformer is in operation. This is referred to as a dielectric function. It also fulfils a mechanical function because the windings rest on it. The third function is to contribute to maintaining an acceptable temperature by creating ducts through which heat is transferred for cooling. As for the oil, its main function is to ensure sufficient dielectric strength and cooling to protect the core and the assembly by filling in the gaps in the insulating materials. Another of its functions is to minimise the contact of cellulose and other materials with oxygen to reduce the risk of oxidation. For the liquid part of insulation, mineral oils have been the most widely used for over a century^1^. However, the controversy over the negative impact of mineral oils on the environment is nowadays a hot topic taken very seriously by environmentalists^2^. This fierce struggle has led researchers to investigate an alternative solution to the shortcomings of mineral oils^3^. One of the alternative solutions proposed to cover the shortcomings of mineral oils is to use vegetable oils. In the last few decades, investigations have made it possible to draw up a wide range of liquid dielectrics from vegetable and synthetic extracts^4^. The various proposals are generally made on the basis of an analysis of the physicochemical, thermal and electrical properties of these liquids in comparison with those of mineral oils. These include acid number, viscosity, flash point and fire point, partial discharge and breakdown voltage, to name but a few.

Talking about the acid index (AI), is an important factor in the degradation of cellulose insulation. However, in a study on the compatibility of vegetable oils as an insulating medium for power transformers, Stefan Tenbohlen^5^ shows that the total AI in natural esters is much higher than in mineral oil. A similar result is reported in the work of Nkouetcha et al.^6^ who make a comparative analysis of this data in mineral oil, palm kernel oil methyl ester and castor oil methyl ester. This work shows that, even after a chemical treatment of these oils, the AI is greater than or equal to that of mineral oil. As regards viscosity, this is one of the most important parameters in the field of power transformer insulation. Heat transfer in liquids is closely linked to the viscosity of the liquid. Liquids with a high viscosity slow down heat transfer and therefore promote heating of the windings. A proposal for vegetable oils as an alternative to mineral oils must therefore take account of their viscosity. Bertrand et al.^7^ carried out an experimental study on the physicochemical characteristics of three vegetable oils compared with those recommended by the ISO 3104 standard. The liquids investigated were the methyl esters of Castor Oil, Sunflower Oil and Rapeseed Oil. Their study showed that all the liquids investigated had a viscosity lower than or equal to that of the standard. However, the results obtained from their experiment show that mineral oils have better characteristics in this respect than vegetable oils. Okafor et al.^8^ conducted work on the study of the viscosity of vegetable oils such as: modified high oleic soybean oil (HOSO), refined low oleic soybean oil (LOSO), acculube oil LB2000 (LB2000) in comparison to mineral oil based emulsion cutting fluid (EC). The results show that the viscosity of all vegetable oils decreases exponentially with temperature, and is significantly higher than that of mineral oil (EC). For the fire point and flash point, there are important characteristics of a liquid insulator. These data are all the more important when the insulation is to be used in an environment with a high thermal concentration, such as a power transformer. Knowledge of this parameter in vegetable oils is important for predicting the maximum thermal stress to be imposed on the insulation without risk of inflammation. These characteristics represent some of the strengths of vegetable oil-based liquid insulation compared with mineral oils. Subburaj et al.^9^ have shown that, the vegetable oils investigated had a flash point and fire point 111% higher than the lower limit recommended by the ASTM D92 standard, compared with 50% for mineral oil. For breakdown voltage, Tenbohlen and Koch^5^ studied the breakdown voltage of the vegetable oil High Oleic 90 Sunflower Oil in comparison with the synthetic ester Midel 7131, the natural ester Envirotemp FR3, and the inhibited mineral oil Nynas Nytro 3000X. Their work shows that the breakdown voltage of vegetable oils is much higher than that of mineral oil. A similar result was demonstrated by Bertrand and Hoang^7^ who investigated the breakdown voltage of vegetable oils such as: castor oil, rapeseed oil and sunflower oil. In a previous work we performed a phase resolution analysis on the propagation of partial discharges in palm kernel oil methyl ester compared to mineral oil^10^. It was concluded that vegetable oil had a better ability to slow down the activity of partial discharges.

Although plant-based liquid insulators have so far proven their effectiveness for possible use in power transformers, a new concept has recently emerged. This is the use of nanofluids based on nanoparticles as a dielectric fluid for high voltage equipment in general and in power transformers in particular. Indeed, at the beginning they were the best way to improve heat transfer^11^. It has been shown that nanofluids have great potential for improving the heat transfer capacity of base fluids as coolants in various applications^12^. Nowadays, nanotechnology has become one of the most exciting and advanced fields of science and engineering^13^. Attention is now focused on how it affects the physicochemical and electrical properties of insulating liquids for power transformers. With regard to the acid number in nanofluids, for example, Asse et al.^14^ investigated the impact of FeO3 nanoparticles on this parameter in palm kernel oil methyl ester. Their work shows that the addition of FeO3 nanoparticles has the negative effect of increasing the AI. They show that, for an addition of nanoparticles at concentrations of 0.10 wt%, 0.15 wt% and 0.20 wt%, an increase in IA of 356%, 317% and 265% respectively is observed. A completely different result is presented in the literature by Hudedmani and Thomas^15^. They conducted a similar experiment with the synthetic ester MIDEL 7131 and nanoparticles such as Ba0.85Ca0.15Zr0.1Ti0.9O3 (BCZT), calcium ferrite (CaFeO3) and eggshell nanomaterials. They show that these nanoparticles have no impact on AI. Regarding viscosity in nanofluids, Madavan et al.^16^ studied the impact of adding *Al*_*2*_*O*_*3*_, BN and *Fe*_*3*_*O*_*4*_ nanoparticles to mineral oil, Honge oil, Neem oil, mustard oil and Punna oil. They concluded that all these nanoparticles degraded the viscosity of the liquids. They showed that viscosity increased with increasing nanoparticle concentration. The same result on viscosity is reported by Sulemani et al.^17^ They show that the addition of the composite of aluminium oxide and zinc oxide (*Al*_*2*_*O*_*3*_ + *Z*_*n*_*O*) to vegetable oils (soya oil, a mixture of sunflower oil and olive oil) increases viscosity by 22%. In terms of flash point and fire point, Sumathi and Rajesh^18^ report that the addition of *TiO*_*2*_, *Al*_*2*_*O*_*3*_ and *MoS*_*2*_ nanoparticles to transformer oil improves these parameters. Tests without additives give a flash point of 142 °C. The results are 157 °C, 142 °C and 148 °C respectively for nanofluids based on *TiO*_*2*_, *Al*_*2*_*O*_*3*_ and MoS_2_ nanoparticles. For the flash point the results are 164 °C, 155 °C and 152 °C respectively for nanofluids based on *TiO*_*2*_, *Al*_*2*_*O*_*3*_ and *MoS*_*2*_ nanoparticles compared to 150 °C in oil without additives. The same experiment carried out by Prasath et al.^19^ with the synthetic ester MIDEL 7131 and titanium nanoparticles (*TiO*_*2*_) shows an increase in the flash point of 2.2%, 3.63% and 4.72% respectively for concentrations of 0.005vol.%, 0.01vol.% and 0.05vol.%. The flash point also increased by 1.71%, 7.16% and 8.50% for the same concentrations. For the breakdown voltage, Šárpataky et al.^20^ studied the impact of the addition of C_60_ nanoparticles on the breakdown voltage of the natural ester MIDEL eN 1204 and the synthetic ester MIDEL 7131. The results showed that the breakdown voltage was increased by 32.5% over the baseline value in both liquids, at an optimum nanoparticle concentration of 0.01% wt./wt. The dielectric strength and endurance ageing test of vegetable oil based nanofluids were studied by Peppas et al.^21^. The results show an increase in BDV for a maximum concentration equal to 0.008% wt./wt. with commercial Fe_3_O_4_ powder and 0.012% wt./wt. for nanofluids with oleate coated colloidal *Fe*_*3*_*O*_*4*_. They also show that the addition of nano-Fe_3_O_4_ has the negative effect of significantly reducing the BDV. With regard to nanofluid ageing, it has been shown that the addition of nanoparticles (*CaCu*_*3*_, *Ti*_*4*_*O*_*12*_, *TiO*_*2*_, *C*_*60*_ etc.) to the insulating oil will improve some of the electrical properties^21^. Du et al.^22^ evaluated the effect of TiO_2_ nanoparticles on the electrical properties of aged insulating mineral oils. They show that the addition of TiO_2_ nanoparticles before ageing increases the BDV by 30% to 40% in contrast to Virgin oil. For the partial discharges, Makmud et al.^23^ studied the activity of partial discharges in nanofluids based on *Fe*_*2*_*O*_*3*_ and palm oil. They show that the discharge inception voltage is 20 kV rms higher in the nanofluid. They also find that the number of PD occurrences is lower at low concentrations than at high concentrations. The same experiment was carried out by Makmud et al.^24^ on the activity of DPs in nanofluids based on palm oil and *Fe*_*2*_*O*_*3*_ and *TiO*_*2*_ nanoparticles. Their work shows that all the types of nanoparticles used significantly improve the inception voltage of DPs and reduce their number of occurrences. However, it also shows that high concentrations of nanoparticles negatively affect the quality of the liquid and promote discharge activity.

As can be seen from the review presented above, the vast majority of studies presenting natural ester oil-based nanofluids as an alternative to mineral oils focus on triglycerides (triesters). In fact, vegetable oils are mainly composed of natural triesters called triglycerides^25^. Triglycerides are molecules containing three ester groups, fatty acids and glycerol. Although triesters offer a number of advantages in terms of dielectric strength and fire resistance, they are subject to other constraints that limit their use in power transformers. Triesters are characterised by a high viscosity, which is a major disadvantage when it comes to heat transfer^26^. Another of its weaknesses is linked to its triple acid chain (R1, R2 and R3), which can be saturated or unsaturated. The large quantity of fatty acids in these oils is likely to increase the rate of oxidation in the insulation system and therefore reduce the life of high-voltage equipment^26^. Among the solutions proposed to improve the performance of triesters in terms of viscosity and heat transfer, work in the literature has shown that a transesterification process has proved very effective in this regard. This protocol is widely used in the production of biofuels and has now been proposed as an alternative solution in the field of high voltage insulation^27–30^. The transesterification process removes two acid chains and changes the oil from a triester to a monoester. This considerably reduces the amount of acid in the oil. Other protocols also make it possible to separate the glycerol from the oil in order to obtain a viscosity that is close to that of mineral oils. Although this method has proved its effectiveness in this field, the literature has not really looked at the application of nanotechnology to monoesters to see what impact it might have on their physicochemical and electrical characteristics. This article focuses on the analysis of the physicochemical and electrical properties of nanofluids based on monoesters of castor oil, iron (FeO3) and zinc (ZnO) nanoparticles. At the end of this work, a summary of some results from the literature on triesters is presented and compared with this work on simple monoesters and monoester-based nanofluids.

## Experimental details

### Preparation of nanofluid samples

**Figure 1:**
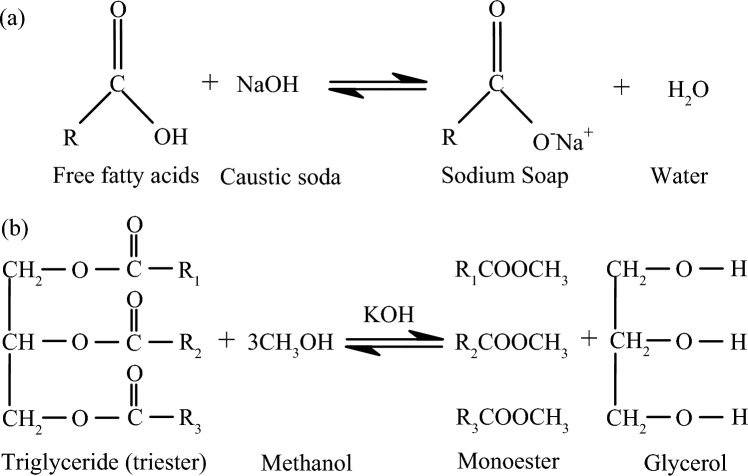
(**a**): Neutralization reaction of free fatty acids, (**b**): transesterification reaction.

**Figure 2:**
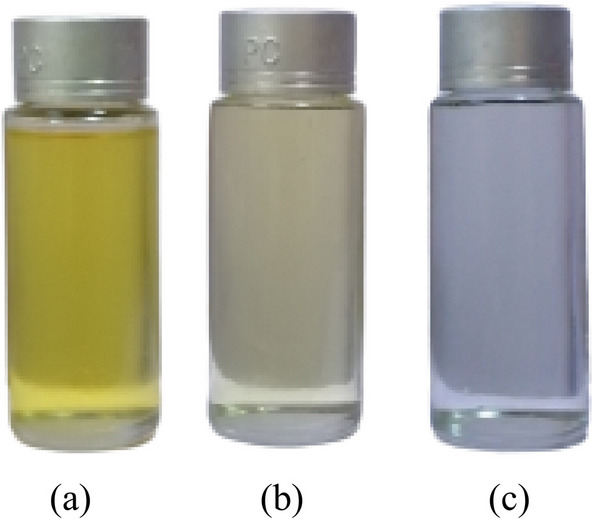
Visual aspect of triesters (**a**, **b**) and monoesters (**c**).

The castor beans were grown and harvested on site, in the Littoral region of Cameroon, using all the methods required by regulations. After harvesting, the seeds were dried and then crushed using a hydraulic press to extract the crude oil. The extracted oils were then purified by degumming followed by a refining operation. The resulting liquid is left to settle for two days and then degummed. Degumming consists of extracting the phospholipids and gums that may become insoluble through hydration. The process consists of adding 20% demineralised water heated to 90 °C to the oil. The whole is mixed slowly at 30 rpm for 40 min. The degummed oil is then recovered by decantation. The next step consists of adding sodium hydroxide (NaOH) to the oil, at a concentration of 14°Be. This step has the effect of reducing the acid number from a free fatty acid neutralisation process (Fig. 1a). The last step consists in reducing the viscosity of the liquid through a transesterification process^31^ (Fig. 1b). The technique consists of adding a basic catalyst such as potassium hydroxide (KOH) to the liquid and then separating by decantation the glycerol responsible for the staling and rapid crystallisation of vegetable oils at room temperature. The monoesters obtained are finally dried at 80 °C for 24 h^32^. Figure 2a,b,c show respectively an example of triesters, triesters after neutralisation of the fatty acids, and monesters of castor oil obtained after transesterification process.

**Figure 3:**
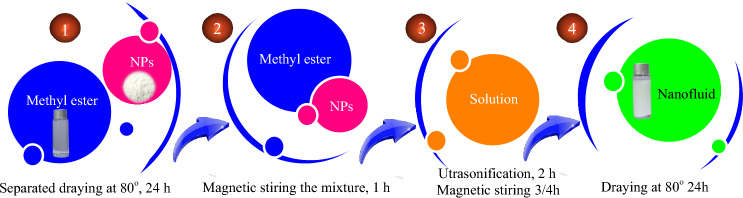
Flow chart for obtaining nanofluids.

**Figure 4:**
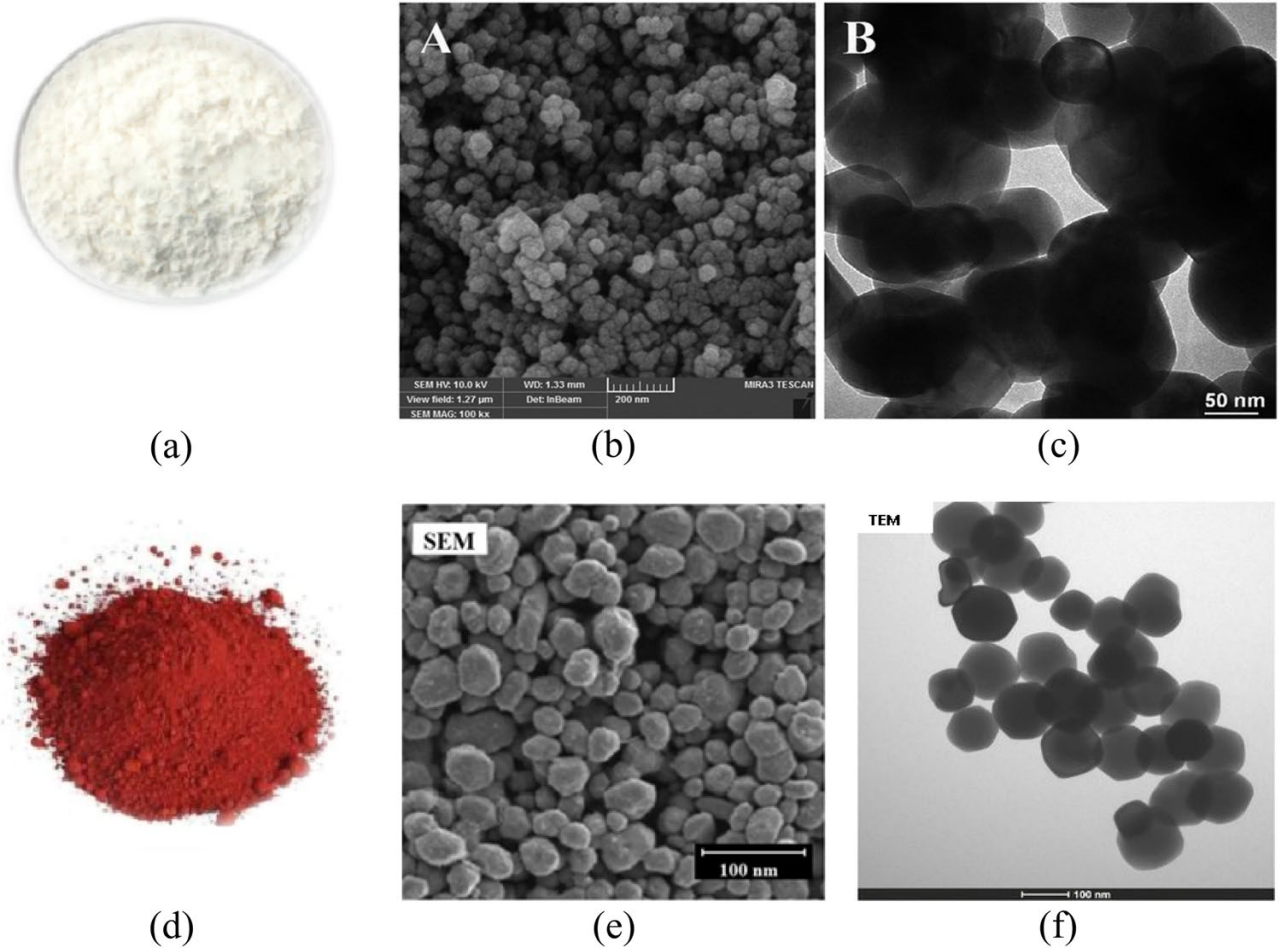
(a, b, c) : Zinc nanoparticles (*Z*_*n*_*O*), (d, e, f) : Iron nanoparticles (*F*_*e*_*O*_*3*_).

The nanofluid samples are obtained by dispersing (*F*_*e*_*O*_*3*_) and (*Z*_*n*_*O*) nanoparticles (NPs) in the liquid as: *E0*=*(COME*+*0* *wt%)*, *E1*=*(COME*+*0.10 wt%)*, *E2*=*(COME*+*0.15 wt%)* and *E3*=*(COME*+*0.20 wt%)*. The choice of concentrations used is based on previous work by the research team^14,33^. The nanoparticles used were ordered from the company Hebei Shengzehong. They are spherical in shape with a mean diameters of 50 nm and 100 nm for *Z*_*n*_*O* and *FeO*_*3*_ respectively. The first step consists of dehumidifying the nanoparticles and esters separately in an oven for 24 hours. The first mixing is done with a magnetic stirrer for 1 hour. The second step consists of homogenising the liquid using an ultrasonic generator (UP-2505) with a power of 150 W and a frequency of 25 kHz for 2 hours as presented in literature^3^. After the sonication phase, the samples were again put under magnetic stirrer for 45 min and dehumidified again for 24 hours. Before each test, the stability test of the samples is carried out by leaving them at rest for a period of 24 hours. Figure 3 shows the flow chart for obtaining nanofluids**.** Figure 4a,d represents the images of the nanoparticles, obtained by simple photography. Figure 4b,e represents the images of the nanoparticles, obtained by a scanning electron microscope (SEM). Figure 4c,f represents the images of the nanoparticles, obtained by a transmission electron microscope (TEM). The stability of the samples was assessed using an ultraviolet spectrometer from Cole-Parmer Ltd, UK (Model 7305, Series 68444). Table 1 shows the stability of the samples over a ten-days.

**Table 1:** Absorbance and stability of samples over ten-days.

Nanofluids $\to$	COME + FeO3			COME + ZnO		
Samples $\to$	E1	E2	E3	E1	E2	E3
Day $\downarrow$	Absorbance (a.u.)					
Day 1	2.118	2.326	2.416	2.051	2.258	2.347
Day 2	2.113	2.298	2.401	2.001	2.186	2.270
Day 3	2.109	2.269	2.386	1.952	2.114	2.193
Day 4	2.104	2.241	2.371	1.902	2.042	2.116
Day 5	2.100	2.213	2.356	1.853	1.970	2.039
Day 6	2.095	2.184	2.341	1.803	1.899	1.962
Day 7	2.090	2.156	2.326	1.753	1.827	1.885
Day 8	2.086	2.127	2.311	1.704	1.755	1.807
Day 9	2.081	2.099	2.296	1.654	1.683	1.730
Day 10	2.078	2.071	2.281	1.605	1.611	1.653
Stability $\to$ (Day 5)	99.15%	95.14%	97.52%	90.35%	87.25%	86.88%
Stability $\to$ (Day 9)	98.11%	89.04%	94.41%	78.25%	71.35%	70.43%

### Measurement of physicochemical properties

The acid index (AI) is measured according to ASTM D6871. The method consists of mixing 10 g of ester with 80 mL of ethanol and leaving it to stir with a magnetic stirrer. While stirring, a KOH solution of concentration [0.1n] previously prepared from 250 ml of ethanol with 1.4 g of KOH is allowed to flow into the mixture. The color indicator used is phenolphthalein. The acid value is thus determined from the volume of solution required to cause the color change. Equation (1)^10^ gives the calculation formula used, where AI represents the acid index (mg KOH/g), V_KOH_ is the volume of KOH flowed (ml) and m_b_ (g) is the mass of oil used.

1 $$AI=5.61\left(\frac{{V}_{KOH}}{{m}_{b}}\right)$$

The viscosities of the samples were measured using a capillary viscometer and according to ASTM D445/ISO3103. The method consists of introducing a quantity of the sample into the viscometer with reference to the standard. The sample is drawn to the prescribed limit and then released. The kinematic viscosity is thus determined from the flow time of the insulating liquid between the viscometer marks according to Eq. (2)^10^. Where *K* is the constancy of the viscometer, t is the flow time of the sample between the two marks, and c the kinetic energy correction.

2 $$\eta =K(t-c)$$

The flash point and fire point of the nanofluid samples were measured according to ASTM D93^34^. The experimental set-up shown in Fig. 4 consists of a hot plate (220 V-5A), an open cup containing the sample, a thermocouple for sampling the sample temperature and a flame applicator. The flash point is identified by introducing a test flame into the opening provided on the sample surface. The flash point temperature is the temperature at which a very brief flame of less than one second duration appears on the surface of the liquid. Similarly, the flash point temperature is the temperature at which a continuous flame appears on the surface of the liquid^35^.

### Breakdown voltage measurement

**Figure 5:**
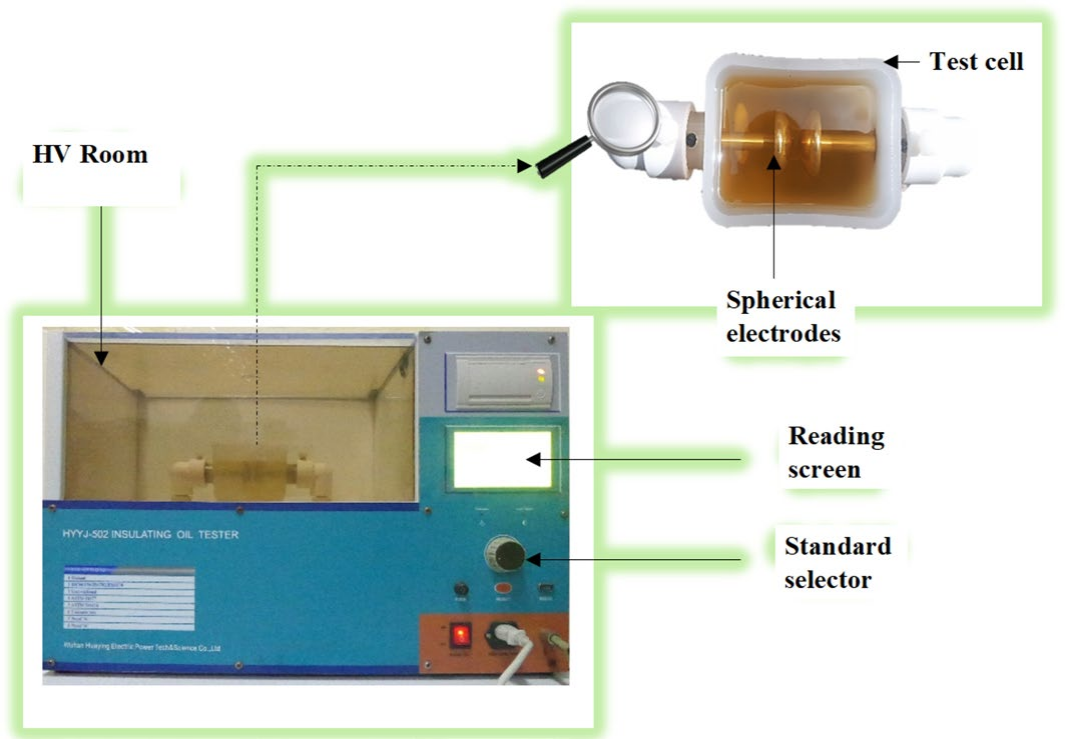
Insulating oil tester (HYYJ-502).

The breakdown voltage is measured using the HYYJ-502 insulating oil tester shown in Fig. 5. The device has a test cell containing spherical electrodes, and an AC voltage up to 100 kV. The tests are performed according to ASTM D1816 with an inter-electrode distance of 1 mm.

### PDIV measurement

**Figure 6:**
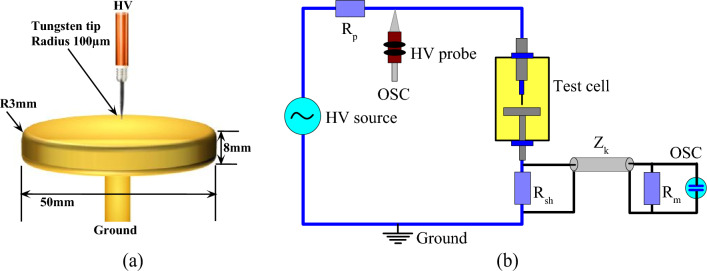
(**a**): Electrode system, (**b**): Set-up.

The device for measuring the partial discharge inception voltage (PDIV) is the same as the one used in the previous work^10^. It consists of a 220 V/50 kV step-up transformer and a control case. The liquid to be tested is contained in a test cell of 500 ml volume containing a tip/plane electrode configuration. The tip electrode is a tungsten needle with a radius of 100 µm and the plane electrode is made of brass. The gap between the electrodes is 2.5 mm. A detailed representation of the electrode configuration is shown in Fig. 6a. The HV transformer and the tip are connected by a 100MΩ current limiting resistor (Rp). The plane is connected to ground via a shunt current sensor (R_sh_). The high voltage is measured with a HV probe and the partial discharges are measured with a DPs sensor. Figure 6b shows the schematic diagram used for the measurement.

**Figure 7:**
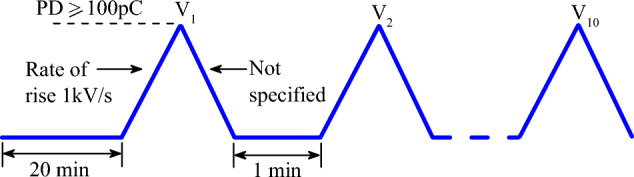
Illustration of the protocol for obtaining PDIV.

The measurement of PDIV is carried out in accordance with the IEC 61,294 standard^36^. This consists of gradually varying the voltage at a rate of 1 kV/s until a discharge with an amplitude greater than or equal to 100 pC is obtained. The voltage giving rise to this charge is considered to be the first value of the IVDP. The voltage is then slowly returned to zero potential and held there for 1 min before the next test. This protocol is repeated ten times to obtain the average PDIV. The recorded currents are studied by the phase-resolved partial discharge (PRPD) models. Figure 7 shows the principle of obtaining the PDIV.

## Goodness of fit test for data analysis

### Anderson–Darling fitting test

The normal distribution of the data is based on the Anderson–Darling test. Initially designed for engineering purposes, the Anderson–Darling test was developed in 1952 by T.W. Anderson and D.A. Darling^37^ as an alternative to other statistical tests for detecting deviation from normality of sample distributions. It is non-directional for one-sample tests and is given by the Eq. (3)^38^.

3 $${{\varvec{A}}{\varvec{D}}}^{2}(\mathbf{x})=-{\varvec{n}}-\sum_{{\varvec{i}}=1}^{{\varvec{n}}}\frac{(2{\varvec{i}}-1)}{{\varvec{n}}}\left[\mathbf{ln}\left({{\varvec{F}}({\varvec{x}}}_{\left({\varvec{i}}\right)})\right)+\mathbf{ln}\left({1-\mathbf{F}({\varvec{x}}}_{\left({\varvec{n}}+1-{\varvec{i}}\right)})\right)\right]$$

where {x(1) < ⋯x(n)} is the sample of size n arranged in ascending order, and F(x) is the underlying theoretical cumulative distribution against which the sample is compared. If AD is greater than the critical value AD_α_ for a given α, the null hypothesis that {x(1) < ⋯x(n)} comes from the underlying distribution F(x) is rejected.

The cumulative distribution function (CDF) of the Weibull distribution is given in Eq. (4) which uses much the same principle**.** The scaling parameter α (kV), which is the characteristic lifetime at which 63.2% of the failure is expected, represents in this context the resistance to flash or fire, and also the resistance to electrical breakdown (BDV). The shape parameter β correlates with the dispersion of the data (*x*). The final decision depends on the P-value calculated according to Eq. (5)^13^. The p-value is compared to the significance level (0.05). If the p-value is less than or equal to 0.05, the null hypothesis that the Weibull distribution fits the data should be rejected, otherwise the null hypothesis that the Weibull distribution fits the data should be accepted.

4 $${\varvec{F}}({\varvec{x}})=1-{{\varvec{e}}}^{-{(\frac{{\varvec{x}}}{{\varvec{\beta}}})}^{\boldsymbol{\alpha }}}$$

5 $${\varvec{p}}-{\varvec{v}}{\varvec{a}}{\varvec{l}}{\varvec{u}}{\varvec{e}}=\left\{\begin{array}{l}\begin{array}{l}{{\varvec{e}}}^{(1.294-5.709{\varvec{A}}{\varvec{D}}+0.019{{\varvec{A}}{\varvec{D}}}^{2})}{\varvec{A}}{\varvec{D}}\ge 0.60\\ {{\varvec{e}}}^{(0.918-4.279{\varvec{A}}{\varvec{D}}+1.380{{\varvec{A}}{\varvec{D}}}^{2})}0.34<{\varvec{A}}{\varvec{D}}<0.60\end{array}\\ \begin{array}{l}{1-{\varvec{e}}}^{(-8.318+42.796{\varvec{A}}{\varvec{D}}-59.938{{\varvec{A}}{\varvec{D}}}^{2})}0.20<{\varvec{A}}{\varvec{D}}<0.34\\ {1-{\varvec{e}}}^{(-13.436+101.140{\varvec{A}}{\varvec{D}}-223.730{{\varvec{A}}{\varvec{D}}}^{2})}{\varvec{A}}{\varvec{D}}\le 0.20\end{array}\end{array}\right.$$

### Kolmogorov-Smirnoff fitting test

The Kolmogorov-Smirnoff KS test of the normality of the data distribution was first introduced by Kolmogorov^39^ and Smirnoff^22^. This test allows a comparison between the distribution of theoretical data and an empirical distribution that would be expected if the data were normal. The KS coefficient can be calculated from Eq. (6)^38^.

6 $${\varvec{K}}{{\varvec{S}}}_{{\varvec{n}}}=\sqrt{{\varvec{n}}}{{\varvec{S}}{\varvec{u}}{\varvec{p}}}_{{\varvec{x}}}\left|{{\varvec{F}}}_{{\varvec{n}}}\left({\varvec{x}}\right)-{\varvec{F}}({\varvec{x}})\right|$$

In this expression, the function F(x) represents the theoretical distribution in x and F_n_(x) the empirical distribution for a sample of size n. The conclusion on the hypothesis is based on the value of KS_n_. For a critical threshold KSα greater than a given value, the null hypothesis that F_n_(x) comes from the underlying distribution F(x) is rejected. The two-sample version of the KS test generalizes to Eq. (7)^38^.

7 $${\varvec{K}}{{\varvec{S}}}_{{\varvec{n}}{\varvec{n}}\boldsymbol{^{\prime}}}=\sqrt{\frac{{\varvec{n}}{\varvec{n}}\boldsymbol{^{\prime}}}{{\varvec{n}}+{\varvec{n}}\boldsymbol{^{\prime}}}}{{\varvec{S}}{\varvec{u}}{\varvec{p}}}_{{\varvec{x}}}\left|{{\varvec{F}}}_{{\varvec{n}}}\left({\varvec{x}}\right)-{{\varvec{F}}}_{{\varvec{n}}\boldsymbol{^{\prime}}}\left({\varvec{x}}\right)\right|$$

In this expression, the functions F_n_(x) and F_n'_(x) represent the empirical cumulative distributions in x for the sample sizes n and n' respectively. The conclusion on the hypothesis is based on the value of KS_n_. For a critical threshold KSα greater than a given value, the null hypothesis that F_n_(x) comes from the underlying distribution F_n'_(x) is rejected. This test has the advantage of being suitable for experimental situations with a small number of samples.

### Shapiro–Wilk fitting test

The Shapiro–Wilk test evaluates the null hypothesis that a sample population (x_1_…x_n_) comes from a normal distribution. It can be evaluated from Eq. (8).

8 $${\varvec{W}}=\frac{{\left(\sum_{{\varvec{i}}=1}^{{\varvec{n}}}{{\varvec{a}}}_{{\varvec{i}}}{{\varvec{x}}}_{({\varvec{i}})}\right)}^{2}}{\sum_{{\varvec{i}}=1}^{{\varvec{n}}}{\left({{\varvec{x}}}_{{\varvec{i}}}-\overline{{\varvec{x}} }\right)}^{2}}$$

With x(i) the i-th order statistic, i.e. the i-th smallest number in the sample. The constants a_i_ are given by Eq. (9).

9 $$({{\varvec{a}}}_{1},\dots ,{{\varvec{a}}}_{{\varvec{i}}})=\frac{{{{\varvec{m}}}^{\boldsymbol{\top }}{\varvec{V}}}^{-1}}{{\left({{{\varvec{m}}}^{\boldsymbol{\top }}{\varvec{V}}}^{-1}{{\varvec{V}}}^{-1}{\varvec{m}}\right)}^{1/2}}$$

where ${\varvec{m}}={{({\varvec{m}}}_{1},\dots {{\varvec{m}}}_{{\varvec{n}}})}^{\boldsymbol{\top }}$ and ${{\varvec{m}}}_{1},\dots {{\varvec{m}}}_{{\varvec{n}}}$ are the expected values of the order statistics of independent and identically distributed random variables sampled from the standard normal distribution, and V is the covariance matrix of those order statistics.

In sum, the normality hypothesis tests of the various statistics mentioned above can be checked by observing the p-value. This parameter is useful for the statistical quantification of a result in the case of a null hypothesis. The objective is to determine whether the null hypothesis is admissible. If it is, the observed result is highly unlikely.

## Experimental results

### Acid index and Kinematic viscosity

**Figure 8:**
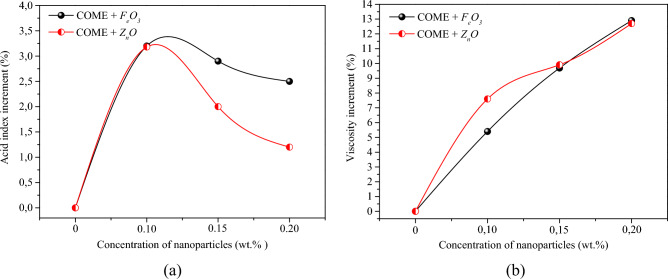
(**a**): Acid index increment, (**b**): viscosity increment.

When an oil-insulated power transformer is put into service, the oxidation process of the system is also initiated. This process is generally favoured by several parameters, including the heat of the winding and the increase in the acid number. Indeed, oxidation due to contamination of the system by foreign materials such as paint, varnish and many others buries a deposit of sludge that can increase with age. This sludge has the negative effect of reducing the heat transfer capacity of the immersed transformer. Knowledge of the impact of the addition of nanoparticles on a parameter such as the acid number is therefore very important. The results of the experiment showed that the addition of nanoparticles at all concentrations had a negative impact on the acid value of the esters. However, the index values obtained remain within the margins set by the IEEE C57.14 standard, which recommends index values below 0.06 (mg KOH/g) for vegetable oils. For the base liquid without nanoparticles, the acid number is 0.041 (mg KOH/g). The addition of iron nanoparticles (*F*_*e*_*O*_*3*_) causes a maximum increment of 3.20% compared to the initial value for a concentration of 0.10 wt%. At this concentration, the same phenomenon is observed with the zinc nanoparticles (*Z*_*n*_*O*) with a maximum increment of 3.18%. We also note that this increment is greater with *F*_*e*_*O*_*3*_ than with *Z*_*n*_*O* for all concentrations. Table 2 summarises the acid number values obtained, and Fig. 8a shows the percentage increments for each concentration. As for the viscosity of the fluid, this is an intrinsic property that measures the characteristic power of liquids to resist the movement of one part of the fluid relative to the other^33^. It is also an important indicator of flow and heat properties, playing an important role in the heat transfer process and particle deposition rate^40^. ISO 3104 recommends less than 11 mm^2^/s at 40 °C for insulating oils for HV/VA distribution transformers. It is therefore important to assess the impact of nanoparticles on this parameter. The results show that the addition of nanoparticles has the negative effect of increasing the viscosity of the enchantments. A similar result is reported by Siddique et al.^41^ who conducted this investigation with Zinc (*Z*_*n*_*O*) nanoparticles. For sample E1, the minimum viscosity increment is 5.4% in *F*_*e*_*O*_*3*_ based nanofluids and 7.6% in *Z*_*n*_*O* based nanofluids. Table 2 shows the values of the viscosity obtained at 40 °C and Fig. 8b shows the increment of the viscosity as a function of the concentration of the nanoparticles.

**Table 2:** Acid number and viscosity of samples.

Samples	Acid index (mg KOH/g)	Increment (%)	St.Dev	Viscosity at 40 °C (mm^2^/s)	Increment (%)	St.Dev
COME + *F*_*e*_*O*_*3*_						
E0	0.0410		0.01	15.06		0.23
E1	0.0423	3.20	0.01	15.87	5.40	0.18
E2	0.0422	2.90	0.02	16.52	9.69	0.22
E3	0.0421	2.50	0.01	17.01	12.9	0.19
COME + *Z*_*n*_*O*						
E1	0.0423	3.18	0.01	16.20	7.60	0.22
E2	0.0418	2.00	0.02	16.55	9.91	0.19
E3	0.0415	1.20	0.01	16.97	12.7	0.23

### Flash point and Fire point

**Figure 9:**
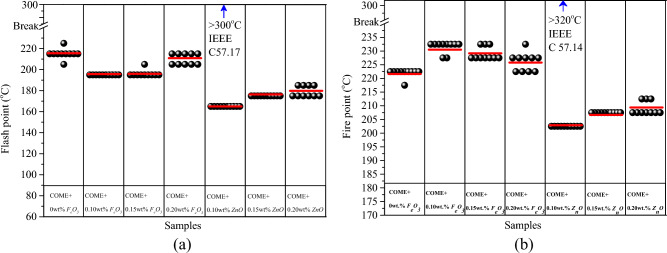
Flash point (**a**) and Fire point (**b**) data for each sample.

When operating a power transformer, the high current draw at the secondary imposes a high thermal stress on the windings and the insulating liquid. The knowledge of the flash point and the fire point of the insulating liquid is therefore important to know the maximum load stress to be imposed on it during operation. Figure 9a,b show the distribution of flash point and fire point data obtained for each nanofluid sample. This representation is based on the "Snap points To Bin" function of the OriginPro software, which rounds the values to the nearest neighbour in order to effectively identify outliers. The mean values are represented by the solid lines. The analysis of the data from this representation shows that, whatever the concentration of zinc nanoparticles (*Z*_*n*_*O*) used, it leads to the degradation of the flash and fire points. However, the opposite phenomenon is observed with iron nanoparticles (*F*_*e*_*O*_*3*_), which tend to improve these two parameters. It can also be seen that all the data are well below that recommended by the IEEE C 57.14 standard which sets the minimum value at 300 °C for the flash point and 320 °C for the fire point. With regard to the flash point in the *F*_*e*_*O*_*3*_ nanofluids, samples E1 and E2 led to a 9% flash point regression compared to the base liquid. Sample E3 has less impact with a maximum regression of 2%. In the ZnO-based samples, the flash point degradation is more considerable. A regression of up to 23% is recorded for E1. The samples E2 and E3 show a regression of 18% and 16% respectively. For the flash point the behaviour is quite different. The results show an increment of the fire point for the *F*_*e*_*O*_*3*_ based samples. The maximum increment is of the order of 4% for E1, 3% and 2% respectively for samples E2 and E3. However, the regression is still effective for the *Z*_*n*_*O*-based samples, but with less impact than in the case of the flash point. A maximum regression of 8.8% is recorded for E1 and 6.88% and 5.63% for E2 and E3.

**Figure 10:**
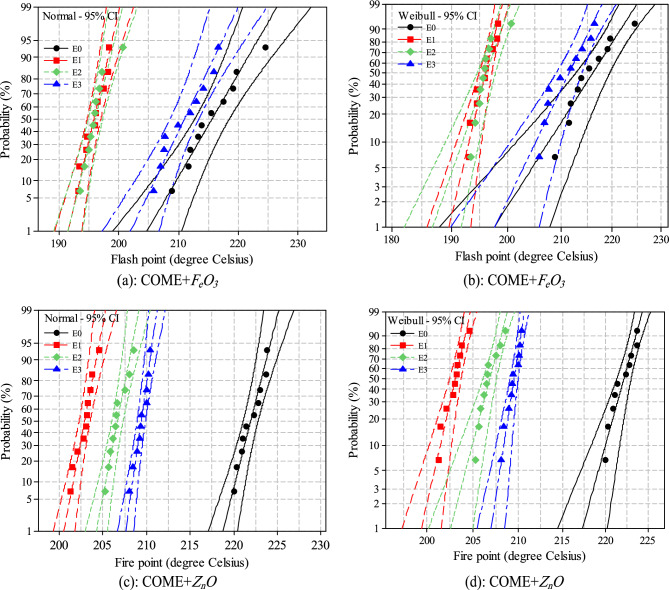
Normal (**a**, **c**) and Weibull (**b**, **d**) distributions of flash points and fire points.

The actual temperature values for the flash and fire parameter showed that the resistance to these phenomena is random in nature and therefore probabilistic. The data was therefore analysed using the Weibull and normal distribution. The lower limit of probability at 1% (P_1%_) estimates the minimum value of temperature at which the Flash or ignition occurs, this value can reveal the reliability level of the insulating liquid. The 50% probability (P_50%_) gives information on the average value of the temperature required to cause flash or fire. Analysis of the distribution of the data according to the two distributions shown in Fig. 10a,b,c,d shows that they follow the normal distribution. This is confirmed by the alignment of the data with the linear fit line.

On the other hand, it can be seen that the P-value for the normality test shown in Table 3 is significantly higher for all samples than the chosen confidence level of 0.05. At 1% risk, there is a maximum flash point regression of 5.6% for the E3 sample based on Iron nanoparticles and 11% for the same concertation in the Zinc nanofluid. For the fire point at the same concentration, we observe an increment of 1% for *F*_*e*_*O*_*3*_ and a regression of 5.7% for *Z*_*n*_*O*. Table 4 summarises the risks at 1%, 10% and 50% (P1%, P50%, P50%) of the flash point for all nanofluid samples. Table 5 summarises the risks at 1%, 10% and 50% (P1%, P50%, P50%) of the fire point for all nanofluid samples.

**Table 3:** Normality test of flash and fire point data.

Samples	Normal		Weibull		Normal		Weibull	
	P-value	Decision	*P*-value	Decision	*P*-value	Decision	*P*-value	Decision
	COME + *F*_*e*_*O*_*3*_				COME + *Z*_*n*_*O*			
Flash point								
*E0*	0.845	*yes*	0.245	*yes*	0.845	yes	0.245	*yes*
*E1*	0.632	*yes*	0.243	*yes*	0.946	yes	0.222	*yes*
*E2*	0.197	*yes*	0.057	*yes*	0.872	yes	0.195	*yes*
*E3*	0.504	*yes*	0.189	*yes*	0.813	yes	0.218	*yes*
Fire point								
*E0*	0.887	*yes*	0.241	yes	0.887	yes	0.241	*yes*
*E1*	0.753	*yes*	0.243	yes	0.570	yes	0.188	*yes*
*E2*	0.844	*yes*	0.229	yes	0.543	yes	0.213	*yes*
*E3*	0.734	*yes*	0.236	yes	0.633	yes	0.199	*yes*

**Table 4:** Probability distributions at 1%, 10% and 50% of flash point.

Samples	Distribution	Flash_P_1%_ (°C)	Increment	Flash_P_10%_ (°C)	Increment	Flash_P_50%_ (°C)	Increment
COME + *F*_*e*_*O*_*3*_							
*E0*	Weibull	198		208		216	
	Normal	205		210		216	
*E1*	Weibull	190	− 04.04	193	− 07.21	196	− 09.25
	Normal	191	− 06.82	193	− 08.09	195	− 09.72
*E2*	Weibull	187	− 05.55	192	− 07.69	196	− 09.26
	Normal	191	− 06.82	193	− 08.09	196	− 09.26
*E3*	Weibull	198	00.00	205	− 01.44	213	− 01.38
	Normal	201	01.52	206	− 01.90	211	− 02.31
COME + *Z*_*n*_*O*							
*E1*	Weibull	162	− 18.18	164	− 21.15	165	− 23.61
	Normal	163	− 20.48	164	− 21.90	165	− 23.61
*E2*	Weibull	174	− 12.12	175	− 15.87	177	− 18.05
	Normal	175	− 14.63	176	− 16.19	176	− 18.52
*E3*	Weibull	176	− 11.11	178	− 14.42	180	− 16.66
	Normal	177	− 13.66	178	− 15.23	180	− 16.66

**Table 5:** Probability distributions at 1%, 10% and 50% of fire point.

Samples	Distribution	Fire_P_1%_ (°C)	Increment	Fire_P_10%_ (°C)	Increment	Fire_P_50%_ (°C)	Increment
COME + *F*_*e*_*O*_*3*_							
*E0*	Weibull	218		220		222	
	Normal	219		220		222	
*E1*	Weibull	227	04.13	229	04.09	231	04.05
	Normal	229	04.57	229	04.09	231	04.05
*E2*	Weibull	222	01.83	226	02.73	229	03.15
	Normal	224	02.28	227	03.18	229	03.15
*E3*	Weibull	216	00.92	222	00.91	226	01.80
	Normal	220	00.45	223	01.36	226	01.80
COME + *Z*_*n*_*O*							
*E1*	Weibull	199	− 08.72	201	− 08.64	203	− 08.56
	Normal	201	− 08.22	202	− 08.18	203	− 08.56
*E2*	Weibull	203	− 06.88	207	− 0591	206	− 07.21
	Normal	204	− 06.85	205	− 06.82	207	− 06.77
*E3*	Weibull	207	− 05.05	208	− 05.45	209	− 05.86
	Normal	207	− 05.48	208	− 05.45	209	− 05.86

### Breakdown voltage

**Figure 11:**
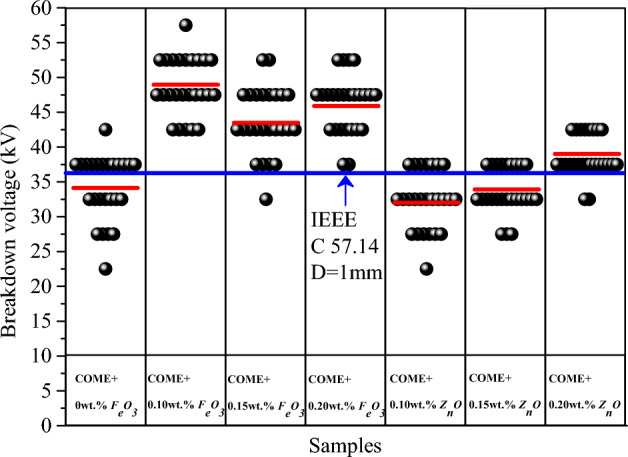
Distribution of the BDV.

Contrary to the negative impact observed in the analysis of the physicochemical properties of the samples, the study of the impact of the nanoparticles on the breakdown voltage showed quite opposite results. From the "Snap point To Bin" representation shown in Fig. 11, it can be seen that all the concentrations used had the effect of increasing the BDV for the *F*_*e*_*O*_*3*_-based nanofluids. The maximum BDV of 49.41 kV was obtained for a concentration of 0.10 wt%. This value is 43% higher than the base liquid, which has a BDV of 34.62 kV, and 33.54% higher than the 37 kV recommended by the IEEE C 57.14 standard. The concentrations of 0.15 wt% and 0.20 wt% caused a 27% and 34% increase in BDV respectively compared to the base liquid of 19.2% and 26.7% compared to the standard. For the *Z*_*n*_*O* nanoparticle samples, only the 0.20 wt% concentration caused a slight increase in BDV of 13.5% compared to the base liquid and 6.2% compared to the standard. The concentrations of 0.10 wt% and 0.15 wt% rather caused a regression of the BDV of 6.5% and 1% compared to the BDV of the base liquid, which itself is already lower than the standard. A similar result was observed by Mashhadzadeh et al^42^. In an experiment conducted with Z_n_O and TiO_2_ nanoparticles, they observed a 27% decrease in breakdown voltage in the *ZnO*-based nanofluid compared to the pure liquid. With the TiO_2_-based nanofluid, on the other hand, they observed an increment of 13%.

**Figure 12:**
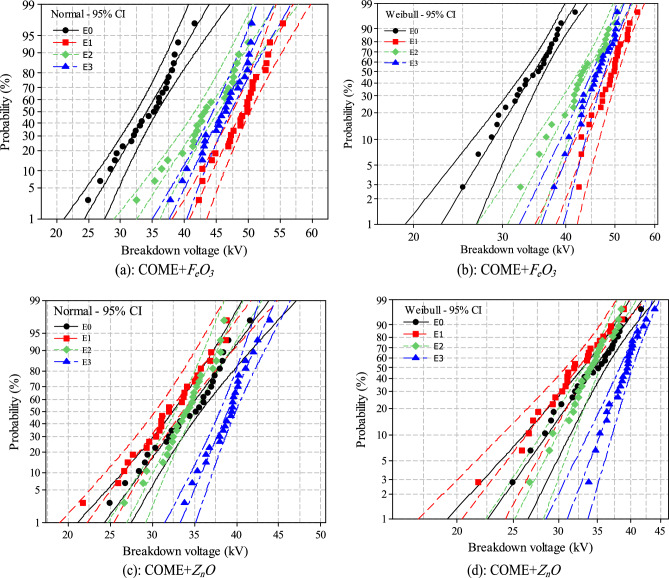
Normal (**a**, **c**) and Weibull (**b**, **d**) distributions of breakdown voltage.

The Weibull and normal distribution of data are the most frequently used probabilistic distributions for analysing BDV data of dielectric liquids^43,44^. It is an effective tool for the prediction of dielectric breakdown at low risk levels. Table 6 presents the summary of the risks at 1%, 10% and 50% (U_1%_, U_10%_ and U_50%_) risk. The analysis of the lower bound probability data (U_1%_) for the *F*_*e*_*O*_*3*_ nanoparticle samples shows increases of 69%, 35% and 58% respectively for the concentrations of 0.10 wt%, 0.15 wt% and 0.20 wt%, compared to the base liquid with a risk value of 22.64 kV. We also note that even though the *Z*_*n*_*O* nanoparticles reduce the BDV for certain concentration values, they still stabilise the lower risk limit (U_1%_). We even observe an increase of 11% and 37% according to the two distributions respectively for the concentrations of 0.15 wt% and 0.20 wt%. The concentration of 0.10 wt% increases the risk to a maximum value of 9.89% according to the Weibull distribution. At 10% and 50% risk, the same phenomenon is observed with nanofluids based on *F*_*e*_*O*_*3*_ nanoparticles, the dielectric breakdown voltage is higher than with *Z*_*n*_*O* and this for all concentrations. The analysis of the data relating to the normality of the breakdown voltage based on the p-value of all the distributions is presented in Table 7. From these we can conclude that the data follows the normal distribution. This is confirmed, as in the case of the fire point, by the alignment of the data to the different fitting lines presented in Fig. 12a,b,c,d.

**Table 6:** Probability distributions at 1%, 10% and 50% of BDV.

Samples	Distribution	U_1%_ (kV)	Increment	U_10%_ (kV)	Increment	U_50%_ (kV)	Increment
COME + *F*_*e*_*O*_*3*_							
*E0*	Weibull	22.64		28.65		34.62	
	Normal	24.33		28.73		34.13	
*E1*	Weibull	38.26	69	44.10	54	49.41	43
	Normal	40.81	68	44.47	55	48.96	43
*E2*	Weibull	30.64	35	37.49	31	44.10	27
	Normal	32.58	34	37.48	30	43.48	27
*E3*	Weibull	35.75	58	41.30	44	46.38	34
	Normal	37.65	55	41.14	43	45.89	34
COME + *Z*_*n*_*O*							
*E1*	Weibull	20.40	− 9.89	26.40	− 7.85	32.45	− 6.54
	Normal	22.26	− 8.50	26.64	− 7.27	32.01	− 6.21
*E2*	Weibull	25.18	11.2	29.89	4.32	34.30	− 0.90
	Normal	27.00	11.0	30.11	4.80	33.92	− 0.61
*E3*	Weibull	31.07	37.2	35.40	23.6	39.30	13.52
	Normal	33.38	37.2	35.91	25.0	39.01	14.29

**Table 7:** Results of the normality hypothesis test for the BDV generated using Minitab software.

Samples	Normal		Weibull		Kolmogorov-Smirnoff		Shapiro–Wilk	
	AD-value	*P*-value	AD-value	*P*-value	KS-value	*P*-value	W-value	*P*-value
COME + *F*_*e*_*O*_*3*_								
E0	0.365	0.409	0.223	0.220	0.121	> 0.150	0.985	> 0.100
E1	0.396	0.344	0.260	0.233	0.122	> 0.150	0.983	> 0.100
E2	0.434	0.278	0.365	0.150	0.129	> 0.150	0.978	> 0.100
E3	0.385	0.337	0.356	0.150	0.094	> 0.150	0.977	> 0.100
COME + *Z*_*n*_*O*								
E1	0.180	0.905	0.177	0.153	0.081	> 0.150	0.989	> 0.100
E2	0.247	0.727	0.268	0.180	0.079	> 0.150	0.985	> 0.100
E3	0.301	0.553	0.316	0.110	0.121	> 0.150	0.989	> 0.100

### Partial discharge analysis

**Figure 13:**
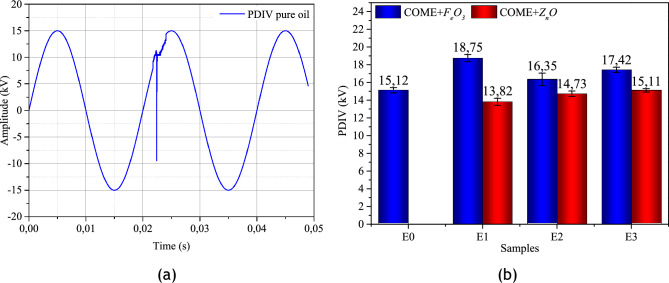
(**a**): Detection of PDIV on the HV probe, (**b**): Average PDIV for each sample.

The measurement of the average inception voltage of the PDs in the nanofluid samples was calculated after their detection according to IEC 61,294. The results show that, as in the case of the breakdown voltage, *F*_*e*_*O*_*3*_-based samples significantly improve the PDIV compared to *Z*_*n*_*O*-based samples. For an inter-electrode gap of 2.5 mm, the PDIV in the pure liquid is 15.12 kV + / − 0.32 in maximum value. In the *F*_*e*_*O*_*3*_-based samples, this value is incremented by 24%, 8.13% and 15.21% for E1, E2 and E3 respectively. In the *Z*_*n*_*O*-based nanofluids, on the other hand, only sample E3 maintains stable PDIV. Samples E1 and E2 lead to a regression of the PDIV of 8.59% and 2.58% respectively compared to the pure liquid. Figure 13a shows an example of the PDIV obtained at the output of the HV probe. Figure 13b shows the summary of the average PDIV over ten measurements in the different samples.

**Figure 14:**
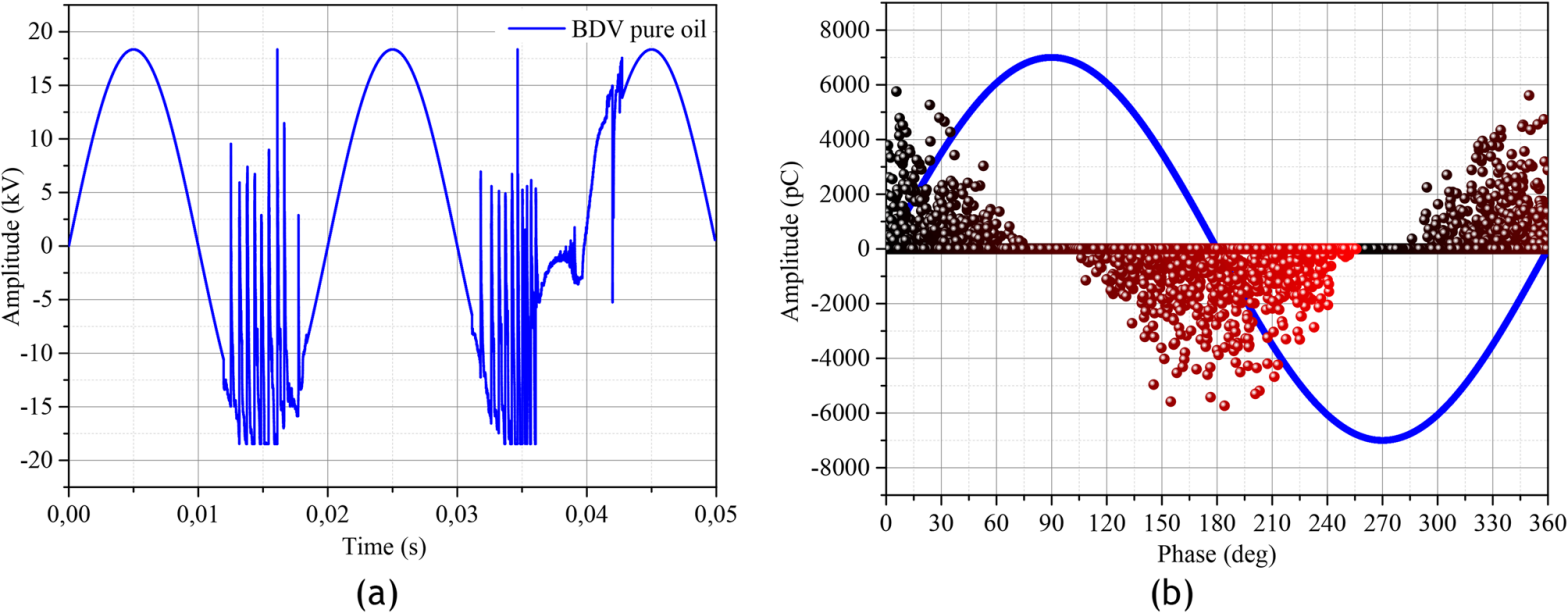
(**a**): Breakdown signal on the HV probe at 1.2*PDIV, (**b**): Partial discharge activity.

The PDs recorded for each sample were analysed by phase-resolved partial discharge (PRPD) models. PRPD is widely used in PD analysis because of the amount of information that can be extracted. The PRPD representation is obtained by recording a continuous flow of PD pulses. Thus, each obtained data can be quantified in terms of phase angle (ϕ), charge magnitude (q) and number of PD occurrences (n). The maintain voltage of the PDs for PRPD analysis was obtained by increasing the voltage to 1.1*PDIV because, for very high voltage values breakdown occurs as shown in Fig. 14a,b obtained for a 100-cycle supply with incrementing the supply to 1.2*PDIV.

**Figure 15:**
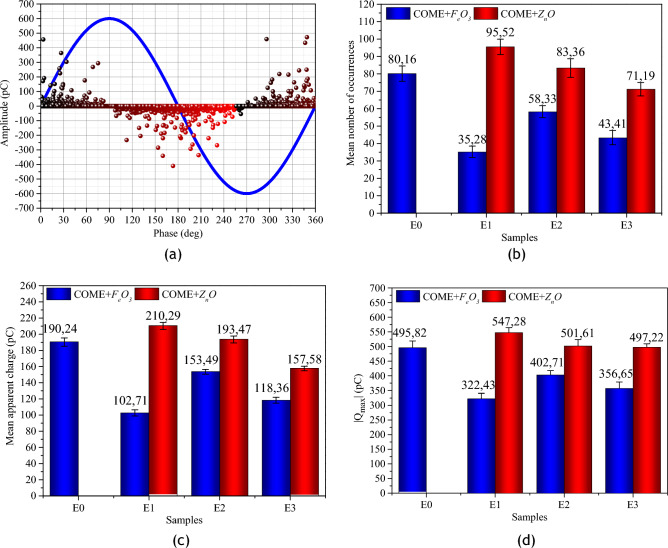
Partial discharge activity in the different samples.

For a voltage increment of 10% above the PDIV, the efficiency of the samples was evaluated by counting the number of occurrences of PDs with a charge greater than 100 pC, evaluating the average charge and the maximum charge of PDs over 2000 cycles. Figure 15a shows an example of the obtained PRPD over 2000 cycles, Fig. 15b shows the average number of occurrences of PDs, Fig. 15c shows the average values of the absolute values of the apparent charges and Fig. 15d the maximum apparent charge in absolute value. The results of this analysis in *F*_*e*_*O*_*3*_-based nanofluids show a regression of the average number of occurrences of 56.00%, 27.23% and 45.85% for samples E1, E2 and E3 respectively. For the *Z*_*n*_*O*-based nanofluids, we observe an increment of 19.16% and 4.00% respectively for samples E1 and E2. For sample E3 there is a regression of 11.20%. For the average charge produced in *F*_*e*_*O*_*3*_ based nanofluids, we observe a regression of 46.01%, 19.21% and 37.72% respectively for samples E1, E2 and E3. In the *Z*_*n*_*O*-based nanofluids, there is an increment of 10.54% and 1.69% for E1 and E2 respectively. For sample E3 we observe a regression of 17.16%. As for the maximum charge in absolute value, we observe a regression of 34.97%, 1.78% and 28.07% respectively for samples E1, E2 and E3 corresponding to *F*_*e*_*O*_*3*_-based nanofluids. For the *Z*_*n*_*O*-based nanofluids, we observe an increment of 10.38% and 1.17%, 0.28% respectively for samples E1, E2 and E3.

### Synthesis of basic characteristics

Tables 8 and 9 present a summary of some results from the literature and from this work on the impact of transesterification on the basic physicochemical and electrical parameters of vegetable oils. These include respectively the triesters and monoesters of: waste cooking oil (WCO, WCOME), ceiba pentandra oil (CPO, CPOME), palm kernel oil (PKO, PKOME) and castor oil (CO, COME). It is clear from this work that the change from triester to monoester has a considerable positive impact on the viscosity of liquids, which is favourable for heat transfer in a power transformer. The significant reduction in the acid number shows that this process is important for anticipating the risks of oxidation in high-voltage equipment and therefore extending its service life. For breakdown voltage, an increment of more than 155% can be observed in certain liquids simply as a result of the switch from triester to monoester. However, important parameters such as fire point and flash point are negatively impacted by this process, with decreases of up to 34% in some liquids. Since the aim of this work was to study the impact of adding nanoparticles to monoesters, Table 9 also gives a comparative summary of the basic parameters in monoester-based nanofluids and those in triester. The results show that parameters such as acid number and viscosity are increased with the addition of nanoparticles. However, the incremental values of these parameters remain well below those of the triesters. This negative trend was also observed with the flash points and changed according to the type of nanoparticles and their concentration in the liquid. We also note that the breakdown voltage is revised upwards and is strongly impacted by the type of nanoparticles and their concentration.

**Table 8:** Comparative results of the literature on triesters and monesters.

Triesters vs monoesters				
A	Standard	Triester	Monoester	
		WCO^27^	WCOME^27^	Impact (%)
(1)	ASTM D7042	40.84	14.19	− 65.26
(2)	ASTM D6871	2.797	0.256	− 90.85
(3)	ISO 2719	269	184	− 31.60
(4)	–	–	–	–
(5)	ASTM D1816	7	30	+ 76.67
		CPO^28^	CPOME^28^	Impact (%)
(1)	ASTM D445	37.37	26	− 30.43
(2)	ASTM D664	29.42	0.087	− 99.70
(3)	ASTM D92	290	279	− 3.79
(4)	ASTM D92	315	308	− 2.22
(5)	ASTM D1816	36	42	+ 16.66
		PKO^6^	PKOME^6^	Impact (%)
(1)	ASTM D1217	26.02	4.87	− 81.13
(2)	ASTM D974	11.22	0.05	− 99.55
(3)	ASTM D92	190	167	− 12.11
(4)	ASTM D92	225	182	− 19.11
(5)	ICE 60,156	37.90	87.68	+ 131.3
		CO^6^	COME^6^	Impact (%)
(1)	ASTM D445	240.28	15.06	− 93.73
(2)	ASTM D6871	3.44	0.05	− 98.54
(3)	ASTM D92	280	183	− 34.64
(4)	ASTM D92	335	218	− 34.93
(5)	ICE 60,156	29.3	74.77	+ 155.3

A: Characteristics, (1): Kinematic viscosity at 40 °C (cSt), (2): Total Acid Number (mg KOH/g), (3): Flash point (°C), (4): Fire point (°C), (5): Breakdown voltage (kV).

**Table 9:** Comparative synthesis of this work on monoesters and monoester-based nanofluids.

Monoester vs monoester based-nanofluids								
A	Standard	Monoester	COME + FeO3		COME + ZnO			
		COME	E1	E2	E3	E1	E2	E3
(1)	ASTM D445	15.06	15.87	16.52	17.01	16.20	15.55	16.97
(2)	ASTM D6871	0.041	0.042	0.042	0.042	0.042	0.042	0.042
(3)	ASTM D93	216	196	196	213	165	176	180
(4)	ASTM D93	222	231	229	226	203	207	209
(5)	ASTM D1816	34.62	49.41	44.10	46.38	32.45	34.30	39.30
Impact (%)								
A			COME + FeO3			COME + ZnO		
			E1	E2	E3	E1	E2	E3
(1)			+ 5.40	+ 9.69	+ 12.9	+ 7.60	+ 9.91	+ 12.7
(2)			+ 3.20	+ 2.90	+ 2.50	+ 3.18	+ 2.0	+ 1.20
(3)			− 9.720	− 9.260	− 2.310	− 23.61	− 18.05	− 16.66
(4)			+ 4.05	+ 3.15	+ 1.80	− 8.560	− 6.77	− 5.860
(5)			+ 43.0	+ 27.0	+ 34.0	− 6.540	− 0.90	+ 13.52

A: Characteristics, (1): Kinematic viscosity at 40 °C (cSt), (2): Total Acid Number (mg KOH/g), (3): Flash point (°C), (4): Fire point (°C), (5): Breakdown voltage (kV).

### Ethical approval

The authors of this papers fully applied the rules of ethics during the experiments and the writing of the paper.

### IUCN policy statement on research involving species at risk of extinction

The castor beans were grown and harvested on site, in the Littoral region of Cameroon, using all the methods required by regulations.

## Discussions

### About flash point and fire point

**Figure 16:**
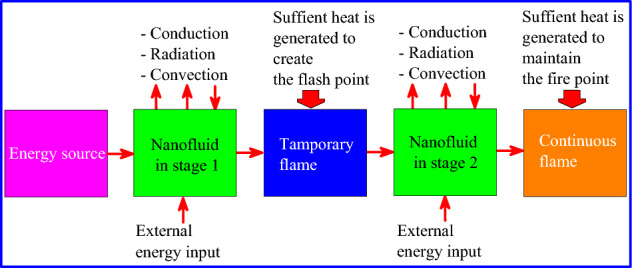
Processes leading to inflammation.

According to the ASTM D93 standard, flash point tests are generally carried out using a container with an orifice open to the air allowing the flame to be applied to the surface of the liquid. Several types of heat transfer are involved in this process, including conduction, radiation and convection. To understand the impact of nanoparticles on the results of the flash point and flash point, the focus is on heat transfer by convection. To make a liquid burn, its surface temperature has to be raised until it produces enough vapor to sustain a flame, a process known as convection. Once the surface temperature produced reaches a critical threshold, an external energy input is required to maintain the flame, and this is known as the fire point. Figure 16 illustrates this process.

**Figure. 17:**
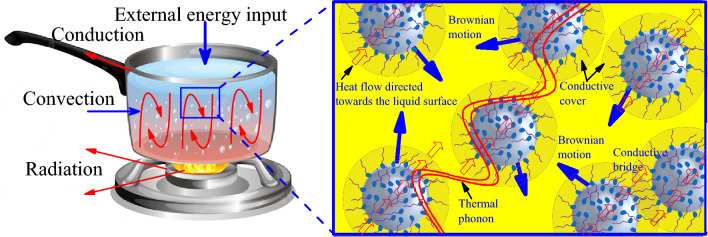
Heat transfer mechanism in nanofluids.

In nanofluids too, heat transfer to the liquid surface involves the phenomenon of micro-convection. Patel et al.^45^ have developed a model for this type of convection in nanofluids. They assume that heat flow through nanofluids involves three parameters, namely conduction through the liquid, conduction through the solid and advection due to the Brownian motion of the particles. There is also agreement in the literature^46,47^ that Brownian motion is the main element contributing to the improvement in thermal conductivity in nanofluids and therefore to the phenomenon of convection. Here four main theories are put forward, namely the Brownian motion of nanoparticles in the fluid, conductive blankets due to the ordering of the fluid molecules around the particle, conductive bridges due to the aggregation of particles or the transmission of heat in ballistic thermal phonons. Figure 17 illustrates this phenomenon.

Patel et al.^45^ consider the liquid and particle phases as continuum. By considering the motion of the particles within the liquid as a flow on a sphere, the overall heat transfer can be expressed as Eq. (10). Experimentally it is shown that the heat transfer enhancement at the nanofluid surface can be given as a proportion of Eq. (11)^45^. In this relationship, *µ*_*p*_ represents the speed of Brownian motion and is given by Eq. (12)^48^.

10 $$q={q}_{m}+ {q}_{p}+{q}_{adv}=\left(-{k}_{m}{A}_{m}{\left(\frac{dT}{dx}\right)}_{m}\right)+\left(-{k}_{p}{A}_{p}{\left(\frac{dT}{dx}\right)}_{p}\right)+\left(\frac{1}{3}h{A}_{p}\Delta T\right)$$

11 $$\%Enhancement=\frac{{k}_{p}}{{k}_{m}}\left(1+c\frac{{u}_{p}{d}_{p}}{{\alpha }_{m}}\right)\frac{{d}_{m}}{{d}_{p}}\frac{\varepsilon }{1-\varepsilon }\times 100$$

12 $${u}_{p}=\left(\frac{2{k}_{B}T}{\pi \eta {d}_{p}^{2}}\right)$$

Here, *A* represents the heat transfer surface area, *k* is the thermal conductivity, *T* the temperature, (*dT /dx*) the temperature gradient of the respective media, m and p represent the quantities corresponding to the liquid medium and the particle respectively. *h* is the convective heat transfer coefficient. *dp* is the particle diameter, d_m_ the molecular size of the liquid. ε the volume fraction of particles in the liquid, *α*_*m*_ is the thermal diffusivity of the liquid, *c* is a constant to be determined experimentally. k_B_ is the Boltzmann constant.

Finally, Eqs. (11) and (12) show that several factors influence the acceleration of heat transfer at the liquid surface. If we look only at the speed of Brownian motion of the particles given in Eq. (12), an initial conclusion can be drawn. The first parameters that can be taken into consideration in this expression are the viscosity of the samples and the temperature applied. Indeed, this speed is proportional to T/η. However, the test temperature is the same for all nanofluid samples. This suggests that samples with a high viscosity reduce the speed of Brownian motion and therefore slow down heat transfer. However, this parameter is very negligible in the context of this study, given that the difference between the dynamic viscosities obtained in *F*_*e*_*O*_*3*_ and *Z*_*n*_*O*-based nanofluids is very small. In these conditions, it is the parameters of the proportionality coefficient that dictate the speed. Furthermore, the average diameter of the nanoparticles used in this study is 50 nm and 100 nm for *Z*_*n*_*O* and *F*_*e*_*O*_*3*_ respectively, i.e. the diameter of *F*_*e*_*O*_*3*_ is on average twice that of ZnO. This implies that the speed of Brownian motion in ZnO-based nanofluids is on average four times that in *F*_*e*_*O*_*3*_-based nanofluids. By transferring this data into Eq. (11), we can see that heat transfer at the sample surface will be greater with *Z*_*n*_*O*. Although other important parameters need to be taken into account in this process, these data give an idea on the difference between the results of the flash point and fire point measurements obtained with the different samples.

### About dielectric performance

**Figure 18:**
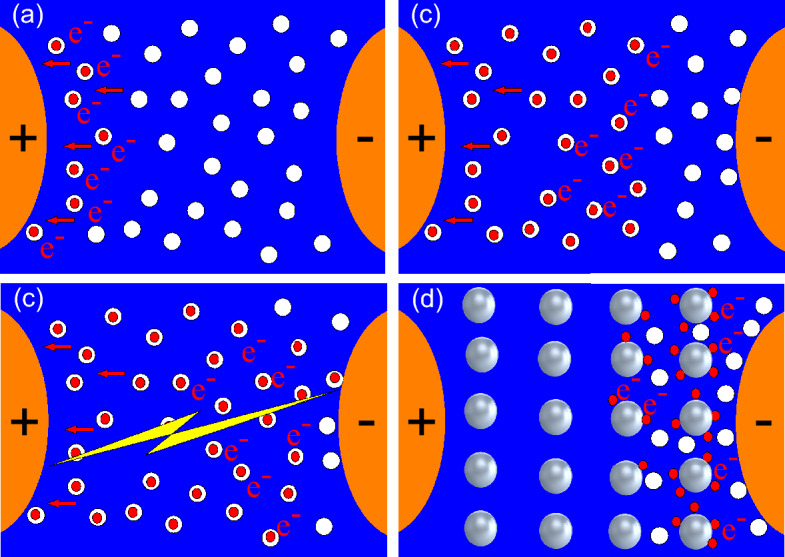
Illustration of the movement of charges in pure oil (**a**, **b**, **c**) and in a nanofluid (**d**).

The increase in breakdown voltage and the reduction in DP activity in the nanofluid can be explained in several ways. Atiya et al^49^, who obtained a similar result with TiO_2_ nanoparticles, claim that, breakdown in insulating liquids is generally manifested by the appearance of streamers resulting from the activity of charges. This reaction generally occurs when the electrical stress is very high. This imposes an electric field whose intensity is greater than the disruptive threshold of the oil molecules, causing ionization. The ionisation process generates ions and free electrons in the liquid. An attractive interaction movement is therefore created between the highly mobile electrons and the positive electrode, leaving behind a zone of net positive charge which propagates towards the negative electrode. Figure 18a,b,c give a progressive illustration of how the movement of charges can be maintained until breakdown (Fig. 18c) in the pure liquid. Positive streamers that make up the electrical discharge during breakdown propagate in four modes closely linked to their speed^49^. The first mode corresponds to discharge activity at relatively low speeds. At this stage, the probability of breakdown is considered to be low and corresponds to U10%. The speed of activity in the second mode is higher and corresponds to an average probability of U50%. The velocities of propagation of the other two modes are greater and correspond to probabilities higher than the average U50%. In the case of nanofluids, the nanopaticules can set themselves up as a barrier hindering the movement of charges from one electrode to another by reducing their speed (Fig. 18d). Depending on the type of nanofluid, this overall average velocity of the charges can be reduced due to the induction phenomenon. Following this logic, the breakdown voltage results at 10% risk (U10%) obtained with the different nanofluids show that *F*_*e*_*O*_*3*_ is better able to hinder the propagation of PDs and streamers in the first and second modes. This result also shows that the electronegativity of *F*_*e*_*O*_*3*_ is higher than that of *Z*_*n*_*O*^42^, making it possible to retain more charge, reduce the number of PDs occurring and reduce the risk of breakdown.

**Figure 19:**
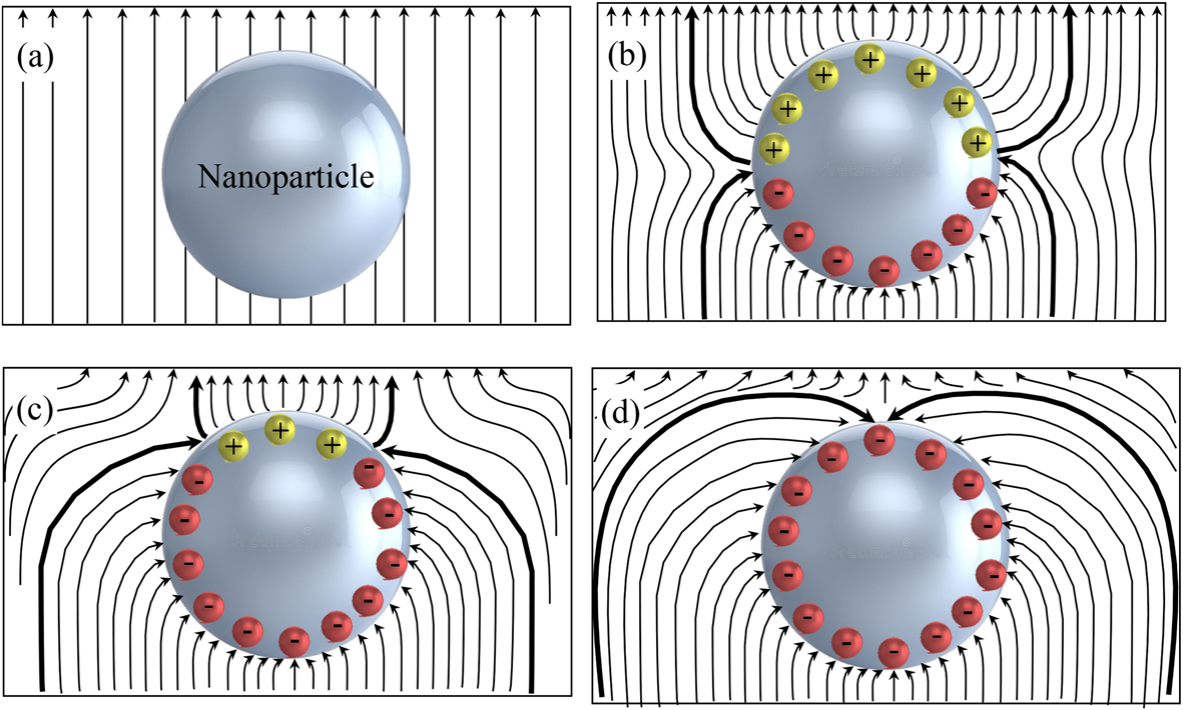
Illustration of the behaviour of nanoparticles.

Concerning the phenomenon of charge induction at the surface of nanoparticles, this occurs when they are subjected to uniform electrical stress^50^. The nanoparticles behave as traps for the charges during their relaxation time. This behaviour reduces and slows down the movement of charges in the liquid, which has the effect of increasing the breakdown voltage. Indeed, under an electric field stress (Fig. 19a), the bulk electrons of conducting nanoparticles such as *F*_*e*_*O*_*3*_ move in the opposite direction to the local electric field. This induces positive and negative charges on opposite sides of the conductor (Fig. 19b) while no space charge is observed in the bulk. Electrons from ionisation or injection move rapidly into the positively charged hemisphere (Fig. 19c) leading to a rebalancing of the charge distribution. This process leads to saturation of the nanoparticle and thus electrons can no longer be trapped (Fig. 19d). This behavior of nanoparticles dispersed in insulating oil is also reported by Koutras et al.^51^. They explain that the surface characteristics of the nanoparticles create traps in the liquid, where free electrons can be easily captured and rapidly released. This phenomenon leads to a considerable reduction in the average energy and mobility of electrons, while the possibility of creating new charge carriers by impact ionisation is further reduced. For semiconductors such as *Z*_*n*_*O*, charge carriers such as holes are the main cause of this type of induction. This mechanism is completely different from those involving dielectric-type nanoparticles, for which we speak instead of electrical polarization involving displacement and rotation polarization. On the other hand, the fact that the *Z*_*n*_*O*-based samples showed a regression of the BDV implies that several other parameters must be taken into account during the process. It is reported that parameters such as relative permittivity and radius in the case of spherical nanoparticles have a considerable impact on the amount of trapped charges and on the relaxation time^50^. Khaled and Beroual^52^ report that the electrical conductivity and dielectric constant of the liquid used and that of the nanoparticles make an important contribution to the final result of the breakdown voltage. It emerges that liquids and nanoparticles with similar parameters will see their breakdown voltage fall while those with completely different parameters will see their breakdown voltage rise. Indeed, the electronic saturation charge of the nanoparticle follows the Eq. (10). The surface charge density of conductive and dielectric NPs can be expressed by Eq. (11)^53^. Equation (10) shows that the saturation charges of the nanoparticle surface are proportional to the square of the radius, which suggests that the radius of the nanoparticles contributes significantly to electron trapping. Equation (11)^54^ shows that if the liquid and the nanoparticles have the same dielectric constant, then all the charges emitted will be transferred to the opposite electrode without any real obstacle. Such behavior is rather favorable to reducing the breakdown voltage and increasing the activity of charges. The charge of a spherical nanoparticle can be calculated according to Sima et al.^55^ by Eq. (12).

10 $${{\varvec{Q}}}_{{\varvec{s}}}=\left\{\begin{array}{c}-\underset{0}{\overset{2{\varvec{\pi}}}{\int }}\left|{{\varvec{\sigma}}}_{{\varvec{s}}}\right|\sqrt{{\left({\varvec{R}}{\varvec{s}}{\varvec{i}}{\varvec{n}}{\varvec{\theta}}\right)}^{2}+{\left({\varvec{R}}{\varvec{c}}{\varvec{o}}{\varvec{s}}{\varvec{\theta}}\right)}^{2}}{\varvec{d}}{\varvec{\theta}}\\ -{{\varvec{R}}}^{2}\underset{0}{\overset{2{\varvec{\pi}}}{\int }}\left|{{\varvec{\sigma}}}_{{\varvec{s}}}\right|{\varvec{d}}{\varvec{\theta}}\end{array}\right.$$

11 $${{\varvec{\sigma}}}_{{\varvec{s}}}=\left\{\begin{array}{c}3{{\varvec{\varepsilon}}}_{1}{{\varvec{E}}}_{0}Cos\theta , Conductive NP\\ 3{{\varvec{\varepsilon}}}_{1}{{\varvec{E}}}_{0}\left(\frac{{{\varvec{\varepsilon}}}_{2-}{{\varvec{\varepsilon}}}_{1}}{2{{\varvec{\varepsilon}}}_{1}+{{\varvec{\varepsilon}}}_{2}}\right)Cos\theta , Dielectric NP\end{array}\right.$$

12 $${\varvec{Q}}\left({\varvec{t}}\right)=\frac{{{\varvec{Q}}}_{{\varvec{s}}}}{{{\varvec{\tau}}}_{{\varvec{p}}{\varvec{c}}}}\frac{{\varvec{t}}}{1+\frac{{\varvec{t}}}{{{\varvec{\tau}}}_{{\varvec{p}}{\varvec{c}}}}},{\varvec{w}}{\varvec{e}}{\varvec{r}}{\varvec{e}}{{\varvec{\tau}}}_{{\varvec{p}}{\varvec{c}}}=\frac{4{{\varvec{\varepsilon}}}_{1}}{\left|{{\varvec{\rho}}}_{{\varvec{e}}}\right|{{\varvec{\mu}}}_{{\varvec{e}}}}$$

where ${{\varvec{\sigma}}}_{{\varvec{s}}}$ is the surface charge density, R is the NP radius, θ is the angle that is occupied by negative charges on NP’s positive hemisphere. ${{\varvec{\varepsilon}}}_{1}$ and ${{\varvec{\varepsilon}}}_{2}$ the oil and NP permittivities, respectively. Eo is the external electric field strength. ${{\varvec{\tau}}}_{{\varvec{p}}{\varvec{c}}}$ is the relaxation time. ${{\varvec{\mu}}}_{{\varvec{e}}}$ is the mobility, ${{\varvec{\rho}}}_{{\varvec{e}}}$ is the electron charge density.

The improvement in PDIV in nanofluids can have several explanations. As with BDV, PDIV is associated with a charge injection process. Electronic motion generally originates at the needle electrode. At relatively low voltages, electrons from the conduction band of the needle must be able to reach its surface and have sufficient energy to overcome the working function of the needle before propagating into the liquid. This process refers to the energy required to pull an electron from the surface of a metal. This implies that the shape and dimensions of the needle electrode play a major role in this process. Kurimský et al.^56^ report that dislocations or asperities of molecular dimensions on the surface of the needle electrode constitute a favourable terrain for the injection of electrons from a metal surface into a dielectric liquid. They claim that dispersing an optimal concentration of nanoparticles in the liquid can homogenise the area of the needle electrode, imposing additional energy to initiate the injection process. This phenomenon is thought to be one of the reasons for the increase in PDIV. In the absence of nanoparticles, when the energy supplied by the applied voltage between the electrodes is sufficient to overcome the work function of the needle electrode, electrons begin to propagate in the liquid. An increase in voltage can lead to charge activity greater than or equal to 100 pC and is referred to as discharge inception. By increasing the voltage further, the number of electrons released by the needle electrode becomes greater and discharge activity increases. Under these conditions, collisions occur between the injected electrons and the oil molecules. This action leads to a transfer of energy, which increases the vibrational energy of the molecules and causes local expansion. If the electrons have sufficiently high energy, they can cause the hydrocarbon bonds to break and the ionisation of the impact. In the case of nanofluids, this process is slowed by the phenomenon of charge trapping, as in the case of BDV.

**Figure 20:**
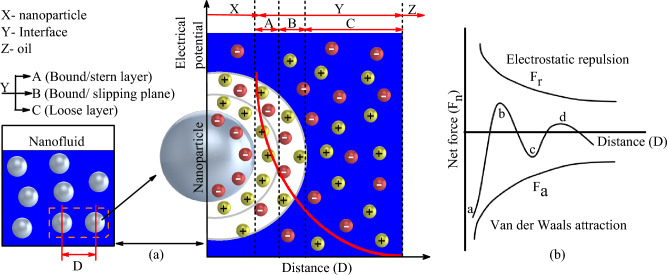
Stability of nanofluids based on (**a**) multicore model of nanoparticle (**b**) DLVO theory.

Returning to the results of his work, the various investigations carried out showed that the dispersion of *FeO*_*3*_ nanoparticles in the liquid produced much more interesting results than *Z*_*n*_*O* in terms of PDIV and BDV. For certain concentrations, ZnO-based nanofluids rather deteriorated the electrical characteristics of the liquid. This result is rather contradictory to the vast majority of works in the literature, which show an improvement in the electrical characteristics of ZnO-based nanofluids^57,58^. This suggests that other important parameters need to be taken into account in this process. Indeed, using a surfactant, Mohamad et al.^59^ carried out a study on the impact of nanofluid stability on PDIV. Their work shows that nanofluids with greater stability have a much higher percentage increase in PDIV, and that the aggregation of nanoparticles in the liquid produces a drop in PDIV. Referring to the stability results of the nanofluids used in this study, it can be seen that FeO3-based nanofluids were more stable than ZnO-based nanofluids. The difference in stability of the two nanofluids used in this study and their impact on breakdown voltage and PDIV can be explained by the DLVO theory presented by Jacob et al.^60^. This theory explains the aggregation of nanoparticles in a fluid on the basis of their multicore model (Fig. 20a). According to this model, the negatively charged particle immediately has a back layer of oppositely charged particles. Beyond this layer is a diffuse layer of weakly bound particles. These different layers form a solid block as the nanoparticle moves through the liquid. The stability of the nanoparticle is closely linked to the forces applied to the charges on this block. DLVO theory refers to the Van der Waals force of attraction *F*_a_ and the electrostatic force of repulsion *F*_*r*_ illustrated in Fig. 20b. The net force of the nanoparticle is the difference of these two forces (*F*_*n*_ = *F*_*r*_*-F*_*a*_). The probability of agglomeration of the particles in a colloid in the liquid is high if this difference in force is negative (see points *a* and *c* Fig. 20b) because in this case there is interaction between the particles. On the contrary, if this quantity is positive (see points *b* and *d* in Fig. 20b) there is repulsion between the particles and they are perfectly dispersed with a much lower probability of agglomeration at point *d*. The results of Jacob et al.^60^ have shown that obtaining maximum stability does not depend on the choice of a high concentration or a low concentration but rather on the search for an optimal intermediate concentration.

Finally, on the basis of the comparison between this analysis and the literature on ZnO-based nanofluids, it emerges that the difference in results between the nanofluids investigated in this work was strongly affected by stability. Since both nanofluids underwent the same dispersion protocol, the main conclusion to be drawn is that the dispersion time depends strongly on the characteristics of the nanoparticles. Furthermore, the choice of a concentration is not random but must be based on a database of experiments linking both the experimental protocol and the characteristics of the nanoparticles.

## Conclusions

The main objective of this paper was to study the impact of the addition of iron (FeO3) and zinc (ZnO) nanoparticles on the physicochemical properties and dielectric performance from castor oil monoester-based nanofluids compared with those from simple monoesters and triesters. From a statistical analysis it was shown that:

- Nanofluids (NFs) based on *F*_*e*_*O*_*3*_ and *Z*_*n*_*O* nanoparticles lead to an increase in the acid number. The maximum increment is 3.2% for a concentration of 0.10 wt% of each NP. However, for all the concentrations used, the acid number remains within the range recommended by the IEEE C 57.14 standard.
- The results also show an increase in viscosity at 40 °C with a maximum increment close to 10 mm^2^/s for both types of NFs with a concentration of 0.15 wt% NPs. All the viscosity values obtained before and after the addition of NPs are well outside the range recommended by the IEEE C 57.14 standard.
- The addition of nanoparticles improves the flash point for *F*_*e*_*O*_*3*_-based NFs and causes a regression for *Z*_*n*_*O*-based samples. However, all concentrations of NPs used lead to a regression of the flash point.
- The average BDV value before the addition of NPs was 7% lower than the value recommended by the IEEE C 57.14 standard. This was raised to more than 43% with *F*_*e*_*O*_*3*_-based NFs, and 13% with *Z*_*n*_*O*-based NFs.
- The partial discharge inception voltage was improved with *F*_*e*_*O*_*3*_ nanoparticles and reduced with *Z*_*n*_*O*. The results of the PRPD analysis of the signal on 2000 clycles show that *F*_*e*_*O*_*3*_ considerably reduced the number of occurrences and the maximum charge of the discharges, unlike *Z*_*n*_*O* for certain concentrations.

## Data Availability

All data and materials are available without restrictions, the corresponding author JLJ is the person to contact in case of need.
